# Effects of Adiposity and Prader-Willi Syndrome on Postexercise Heart Rate Recovery

**DOI:** 10.1155/2013/384167

**Published:** 2013-05-22

**Authors:** Diobel M. Castner, Daniela A. Rubin, Daniel A. Judelson, Andrea M. Haqq

**Affiliations:** ^1^Department of Kinesiology, California State University, Fullerton, 800 North State College Boulevard, KHS-236, Fullerton, CA 92831, USA; ^2^Department of Pediatrics, University of Alberta, Edmonton, AB, Canada T6G 2R7

## Abstract

Heart rate recovery (HRR) is an indicator of all-cause mortality in children and adults. We aimed to determine the effect of adiposity and Prader-Willi Syndrome (PWS), a congenital form of obesity, on HRR. Sixteen children of normal weight (NW = body fat % ≤85th percentile, 9.4 ± 1.1 y), 18 children with obesity (OB = body fat % >95th percentile, 9.3 ± 1.1 y), and 11 PWS youth (regardless of body fat %; 11.4 ± 2.5 y) completed peak and submaximal bike tests on separate visits. HRR was recorded one minute following peak and submaximal exercises. All groups displayed similar HRR from peak exercise, while NW (54 ± 16 beats) and OB (50 ± 12 beats) exhibited a significantly faster HRR from submaximal exercise than PWS (37 ± 14 beats). These data suggest that excess adiposity does not influence HRR in children, but other factors such as low cardiovascular fitness and/or autonomic dysfunction might be more influential.

## 1. Introduction

Heart rate recovery (HRR) is the rate at which heart rate declines from the end of exercise to a predetermined time point during recovery, typically one minute after exercise [1, 2]. HRR depends on an immediate parasympathetic nervous system reactivation followed by sympathetic activity withdrawal [3]. After submaximal exercise, the withdrawal from sympathetic activity typically takes place 30 seconds into recovery [2, 4]. In contrast, increased sympathetic activity following peak intensity exercise may continue for approximately one to three minutes, delaying HRR [2, 5, 6]. A slower HRR has been related with lower cardiovascular fitness, thereby indicating an increased risk of cardiovascular disease [7]. Therefore, HRR has been suggested as a noninvasive method for cardiovascular risk assessment in both children and adults [3, 8]. 

 Body fat is positively associated with risk for cardiovascular disease in children [9] and adults [10]. As HRR is an indirect measure of cardiovascular disease risk, studies in adults have shown that indices of obesity are strong predictors of HRR following exercise [3, 11]. Dimkpa and Oji showed that in healthy adults, obesity surrogates negatively correlated with HRR following submaximal intensity exercise [11]. Similarly, Singh and colleagues demonstrated that overweight youth experienced a slower one-minute postexercise HRR than healthy weight youth after a maximal protocol followed by one-minute cool-down period [8]. Moreover, Dangardt and colleagues suggested that children with obesity displayed a significantly lower vagal activity at rest compared to overweight and lean controls [12]. Though these studies related body fat and HRR, no research has investigated HRR from aerobic exercise in children with excessive adiposity (i.e., those classified as obese, not overweight) compared to lean controls.

 In addition, measuring HRR in children predisposed to excessive adiposity (e.g., individuals with Prader-Willi Syndrome) can also reveal particular mechanisms that affect postexercise HRR response. Prader-Willi Syndrome (PWS) is the best characterized genetic cause of childhood obesity [13]. PWS is characterized by hypotonia, hyperphagia, abnormally high adiposity, hypogonadism, and lack of normal growth hormone production [14]. Past work investigating autonomic nervous system (ANS) function in youth with PWS presents equivocal results [15–18]; therefore, those with PWS are a particularly unique comparison group to nonsyndromal children as HRR may be related to congenital adiposity and/or syndrome-related ANS dysfunction.

 Stinted HRR immediately following peak exercise reveals deficits in parasympathetic reactivation [2, 5, 6], while stinted HRR from submaximal exercise reveals deficiencies in either parasympathetic reactivation or sympathetic withdrawal [2, 4]. Thus, studying HRR response from peak and submaximal exercise provides information about different ANS regulatory mechanisms. This study investigated whether adiposity and PWS influenced postexercise HRR from two different exercise intensities.

## 2. Methods

### 2.1. Participants

Sixteen (7 M and 9 F) children of normal weight (NW = body fat percentage ≤85th percentile for age and sex; BMI z-score: −0.1 ± 0.6; age: 9.4 ± 1.1 y), 18 (12 M and 6 F) children with obesity (OB = body fat percentage >95th percentile for age and sex; BMI z-score: 2.0 ± 0.5; age: 9.3 ± 1.1 y) [19], and 11 (8 M and 3 F) youth diagnosed with PWS (BMI z-score: 1.7 ± 0.7; age: 11.4 ± 2.5 y) participated in this study. This study was approved by the Institutional Review Boards from California State University, Fullerton, Children's Hospital of Orange County, and the United States Army Medical Research and Materiel Command. Written informed assent and consent were obtained from all participants and parents prior to participation. Children with diabetes mellitus type 2, confirmed pregnancy, or those unable to participate in moderate to vigorous physical activity were excluded from participation.

### 2.2. Experimental Design

Participants completed two visits separated by two to 14 days. During visit one, all participants were measured for anthropometrics, body composition, resting heart rate (HR), and blood pressure (BP); subjects then completed a graded exercise test. During visit two, participants performed a discontinuous submaximal test. Participants were instructed not to engage in physical activity (i.e., sports games, bike rides, physical education, etc.) for a minimum of 24 hours before exercise testing. In addition, participants were provided with a standardized breakfast that contained no caffeine to consume two hours prior to visit arrival. This study was part of a larger research effort devoted to examining the physiological and hormonal responses to exercise in youth with PWS.

### 2.3. Visit One

#### 2.3.1. Medical Screening

Parents of participants completed a medical history questionnaire regarding their child's health and participation in moderate to vigorous physical activity. Children without PWS completed the Pubertal Developmental Scale [20] to assess pubertal status. PWS youth underwent a full health screening by a physician to determine contraindications for exercise testing and participation in the study, as well as Tanner stage determined by breast, genital, and pubic hair development.

#### 2.3.2. Anthropometric and Physiologic Measurements

Participants removed shoes before all measurements. Body mass was measured using a digital scale (ES200L, Ohaus, Pinewood, NJ) while participants wore a t-shirt and shorts. Height was measured after participants inhaled using a wall-mounted stadiometer (Seca, ON, Canada). Waist circumference was measured following NHANES guidelines at the top of the participant's iliac crest at the end of exhalation [21]. Total body fat percentage was determined using a whole body dual energy X-ray absorptiometry (DXA) scan (GE Healthcare, GE Lunar Corp., Madison, WI). Female participants who had their first menses were required to complete a pregnancy test prior to completing the DXA scan. Resting HR, measured via telemetry (Polar USA, Lake Success, NY), and resting BP, measured via aneroid sphygmomanometer (Diagnostix 752, American Diagnostic Corporation, Hauppage, NY), were recorded following five minutes of seated rest [22]. 

#### 2.3.3. Peak Graded Exercise Test

Participants completed the McMaster protocol, a cycling protocol tailored to height and sex [23], on a cycle ergometer (Corival Pediatric, Lode B.V., the Netherlands; Technogym Bike Med, Technogym USA Corp., Seattle, WA) suited to the child's stature. The test consisted of two-minute stages at incremental workloads until the participant reached volitional exhaustion, failed to sustain the desired workload [24], requested to stop, stood up on the bike pedals, or experienced fatigue-related symptoms [25]. 

Relative peak power output was computed by dividing the test termination load by the participant's lean body mass obtained from the DXA scan. BP was measured at rest and the end of exercise. HR was recorded at test termination and one minute following exercise. HRR was determined by computing a HRR value (HRRV) calculated as the difference between test termination HR and HR recorded after one minute after exercise [1]. One-minute postexercise HRR is the most commonly used methodology to assess recovery heart rate in children [1–3, 6, 8]. 

### 2.4. Visit Two

Participants completed a discontinuous submaximal test consisting of ten two-minute cycling intervals each separated by one minute of rest. The resistance setting was based on the HR obtained during the McMaster protocol and chosen to elicit a HR ≥160 bpm throughout. This intensity was chosen based on a previous study that used a discontinuous protocol in youth of normal weight and obesity to assess counterregulatory hormonal responses to acute exercise [26]. BP and HR were measured and recorded using the same procedures as visit one. 

### 2.5. Data Processing and Analysis

One-way analysis of variance (ANOVA) tests were initially conducted to determine group differences for participant characteristics and all exercise responses. HRRV was used to compare HRR among groups for both exercise intensities. ANOVAs were then conducted to determine group differences for HRRV for each exercise intensity. In case of significant group differences, Tukey's post hoc tests were used to determine pair-wise differences. Significance level for all statistical analyses was set at *P* < 0.050. IBM SPSS Statistics 19.0 for Windows (SPSS, Inc., Chicago, IL) was used for the statistical analysis.

## 3. Results

No sex differences were found between and within groups for HRR; therefore, all children were analyzed together. As expected, OB and PWS displayed a significantly greater total body mass, waist circumference, trunk body fat percentage, and total body fat percentage compared to NW. In contrast, NW had significantly greater lean mass percentage than their counterparts; there was no difference in lean mass percentage between OB and PWS (Table 1). All groups had a similar absolute peak workload, peak SBP, and peak DBP, while youth with PWS exhibited a lower relative peak power output (expressed per kg of lean body mass) and peak HR than NW or OB (see Table 2), who had similar relative peak workloads and HR. Absolute submaximal workload was also similar amongst groups; however, PWS had a lower relative power output than NW only during submaximal exercise (see Table 3)—OB children were not significantly different from either PWS or NW. When expressed as a percentage of peak relative workload, all groups worked at a similar aerobic effort during submaximal exercise (Table 3). All groups had significantly different mean submaximal exercise HR responses, with NW having the highest and PWS having the lowest (see Table 3). When expressed as a percentage of peak HR, the HR response during submaximal exercise was significantly lower in OB compared to NW; PWS was similar to both groups (Table 3). OB also had a significantly higher SBP in response to submaximal exercise compared to NW, and PWS had a similar response to both groups. All groups had a similar submaximal exercise DBP.

**Figure 1:**
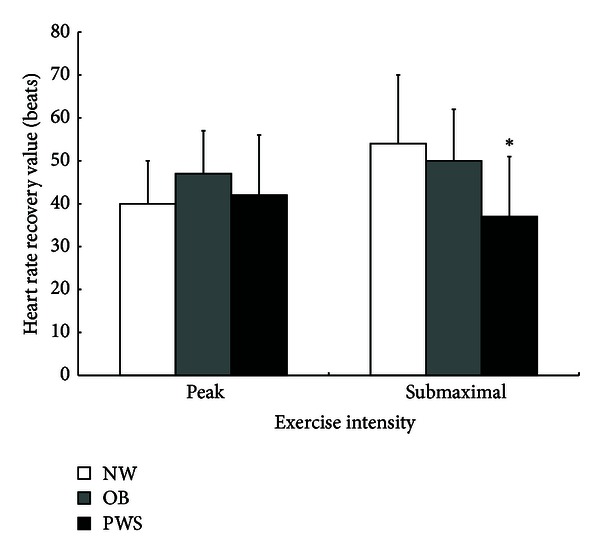
Postexercise HRRV (beats) by group and exercise intensity, presented as mean ± SD; *P < 0.050.

There were no significant group differences for HRRV from peak exercise. For submaximal intensity exercise, youth with PWS had a significantly lower HRRV compared to both NW and OB, who had similar HRRV (Figure 1). 

## 4. Discussion

The results of this study showed that the decline in heart rate following exercise was not dependent on excessive adiposity. All groups had similar HRR in response to peak exercise. The only difference observed was in youth with PWS who displayed a significantly slower HRR following submaximal intensity exercise compared to other children. Two possible mechanisms explain the lower HRR following submaximal exercise in PWS: cardiovascular fitness and/or autonomic abnormality. 

Children of normal weight and those with obesity but without PWS had similar recovery responses following both peak and submaximal aerobic exercises. Therefore, the results of this study contradicted the results of an earlier study by showing that increased adiposity did not affect postexercise HRR in children with nonsyndromic obesity [8]. However, there was a key methodological difference between the previous study [8] and the present study. The present study measured passive (i.e., no cool down, seated) HRR immediately following test termination. By doing so, postexercise HRR was not affected by continued, voluntary sympathetic activation (which occurs during active cool down) and better isolated true recovery rate. 

Another factor that could have influenced the HRR results of this study is cardiovascular fitness. Higher levels of cardiovascular fitness have been positively associated with a faster HRR following exercise [27–31]. Adults with high levels of physical activity have shown to either sustain or improve HRR over 20 years compared to adults with low levels of physical activity [28]. In addition, HRR improved after exercise training and a hypocaloric diet in obese children [31]. The fact that cardiovascular fitness is related to HRR may partially explain the results of this study. Both participants with obesity and those of normal weight were similarly fit (i.e., peak W·kg LBM^−1^, peak HR, and peak estimated [23] VO_2peak_ (13.9 ± 1.0 versus 13.6 ± 1.2 mL·kg LBM^−1^·min)). In contrast, those with PWS were significantly less fit compared to children without PWS (i.e., lower peak HR, lower W·kg LBM^−1^and lower estimated [23] VO_2peak_ (12.4 ± 1.3 mL·kg LBM^−1^·min)). Previous work showed poor cardiovascular fitness and low physical activity level in PWS [32, 33]. Poor cardiovascular fitness in PWS might be best explained by physiological characteristics inherent to the syndrome including hypotonia [32], reduced knee flexor and extensor muscle strength [34], and/or muscle fiber size deficiency and atrophy [35]. Therefore, the lower HRR after submaximal exercise in those with PWS compared to the other children possibly indicates that the low cardiovascular fitness in those with PWS was related to the slow HRR [28, 31, 36]. 

In addition, it is possible that HRR in PWS may have also been influenced by altered ANS function. A review by Haqq et al. suggests that in obesity there is an increased sympathetic activity as well as reduced parasympathetic activity [37]. This mechanism is not so clear in PWS, although it appears that PWS presents similarities with an autonomic disorder called familial dysautonomia where more sympathetic neurons are affected than parasympathetic neurons [37]. Richer and colleagues investigated a small cohort of children with PWS and controls to determine possible differences in ANS function. Postganglionic sympathetic function was evaluated through Quantitative Sudomotor Axon Reflex testing (sweat volume), cardiovagal testing (HR response to deep breathing), and pupillary response (HR and BP responses and HR variability spectral analysis to head-up tilt). Children with PWS exhibited a trend towards lower total sweat volume, a smaller HR increase with head tilt-up, and lower low frequency power HR variability at rest and with head-up tilt compared to the controls indicating possible impaired sympathetic function [15]. 

The results of the present study showed a lower peak HR in PWS perhaps related to lower muscular work capacity or sympathetic stimulation. However, the lower HRR following submaximal exercise in those with PWS compared to nonsyndromal children may indicate a delayed sympathetic withdrawal and impaired function as suggested by Richer and colleagues [15]. In terms of peak exercise responses, it is possible that the chosen protocol limited the capacity to fully assess ANS function, and therefore no differences among groups were obtained. Past studies suggested that after peak intensity exercise HRR is delayed due to sympathetic activity lasting one to three minutes into the recovery period. The present study measured HRR only during the first minute after exercise, and this is a limitation of the study design and results [2, 5].

In addition, the participants with PWS were significantly older than nonsyndromal obese controls, which might explain the deterioration in ANS function [38]. Seven youth with PWS presented Tanner stages I–III, while two youth presented stage IV and one stage V. In comparison, all nonsyndromal children presented Tanner stages I through III based on their self-report. Follow-up analyses were done to determine differences among groups in HRR including only participants with Tanner stages I through III. Youth with PWS (*n* = 7) still presented a significantly slower HRR following submaximal intensity exercise compared to NW (*n* = 16). However, OB (*n* = 17) responded similarly to both groups following submaximal exercise. Similar to the previous analyses, no group differences were observed following maximal intensity exercise. It is possible that those youth with PWS in later stages of puberty experienced more ANS deterioration, exacerbating the HRR differences with the nonsyndromal obese controls. Regardless, youth with PWS still showed a delayed recovery from submaximal intensity exercise compared with normal weight controls, indicating possible ANS dysfunction. It is necessary for future studies to account for pubertal status as a screening criterion to better assess whether the differences in HRR are related to ANS function. Lastly, recording blood pressure at the end of the recovery period is another measurement that could have helped assess the ANS function in response to exercise. Overall, the results of this study suggest that ANS dysfunction in youth with PWS could influence HRR following submaximal intensity exercise. 

Having participants with PWS complete these protocols presented barriers. Most of these limitations were related to the physiological (lower than normal muscle force capacity, lack of stamina, and lower than normal motor proficiency), psychological (mild to severe mental retardation and mental rigidity), and behavioral (attention deficit, temper tantrums, and inability to accept change) characteristics of PWS [13, 14, 32, 33, 39]. Even with these difficulties, those with PWS were able to complete a graded peak exercise protocol. It was verified that the submaximal effort was similar between those with and without PWS by calculating the percentage of relative power output and heart rate compared to peak effort. Therefore, the present study results were not affected by the exercise protocols. The present study lacked comparison groups with low and high cardiovascular fitnesses, a comparison that would have isolated the effect of cardiovascular fitness on HRR. However, a study with such research design may not be feasible given that inherently PWS presents poor cardiovascular fitness.

## 5. Conclusion

As demonstrated by the similar recovery rate in children of normal weight and those with obesity, adiposity did not influence HRR following peak or submaximal exercise. Youth with PWS showed a low cardiovascular recovery capacity, suggesting that poor cardiovascular fitness and/or an altered ANS function may impact HRR. 

## Figures and Tables

**Table 1:** Participant demographics and characteristics, presented as frequencies and mean ± SD.

	NW	OB	PWS
Frequency	16	18	11
Male/female	7/9	12/6	8/3
Age (y)	9.4 ± 1.1^‡^	9.3 ± 1.1^‡^	11.4 ± 2.5
Pubertal stage (Tanner)			
I	9	9	3
II	4	5	2
III	3	3	2
IV	0	0	2
V	0	0	1
Body mass (kg)	32.76 ± 5.58	53.18 ± 13.31*	57.70 ± 22.67*
Body mass index z-score	−0.1 ± 0.6	2.0 ± 0.5*	1.7 ± 0.7*
Waist circumference (cm)	60.7 ± 4.4	85.3 ± 11.9*	84.1 ± 17.8*
Trunk fat (%)	16.7 ± 5.1	42.9 ± 7.8*	43.5 ± 8.4*
Fat mass (%)	18.7 ± 4.6	41.2 ± 7.5*	43.4 ± 8.2*
Lean mass (%)	78.1 ± 4.3	57.0 ± 7.2*	54.8 ± 7.8*
Lean mass (kg)	25.32 ± 3.91	29.31 ± 4.42	30.91 ± 11.92
Resting heart rate (bpm)	86 ± 10	80 ± 9	81 ± 13
Resting SBP (mm Hg)	99 ± 9	106 ± 11	113 ± 12*
Resting DBP (mm Hg)	60 ± 12	69 ± 11*	68 ± 6

Values are significant at *P* < 0.050; *different than NW; ^‡^different than PWS. Pubertal status was not obtained from one OB and one PWS.

**Table 2:** Responses to peak intensity exercise, presented as mean ± SD.

	NW	OB	PWS
Absolute peak power output (W)	96.7 ± 16.9	105.9 ± 21.4	94.6 ± 55.7
Relative peak power output (W·kg LBM^−1^)	3.8 ± 0.5^‡^	3.6 ± 0.7^‡^	2.9 ± 0.7
Peak heart rate (bpm)	186 ± 12^‡^	190 ± 14^‡^	167 ± 18
Peak SBP (mm Hg)	129 ± 19	142 ± 18	126 ± 20
Peak DBP (mm Hg)	63 ± 9	67 ± 13	69 ± 9

Values are significant at *P* < 0.050; ^‡^different than PWS.

**Table 3:** Responses to submaximal intensity exercise, presented as mean ± SD.

	NW	OB	PWS
Absolute submaximal power output (W)	67.5 ± 14.0	69.0 ± 14.8	64.8 ± 31.3
Relative submaximal power output (W·kg LBM^−1^)	2.7 ± 0.4	2.4 ± 0.4	2.1 ± 0.3*
Percentage of peak relative workload (%)	70.9 ± 15.0	65.9 ± 10.5	71.5 ± 10.5
Submaximal exercise heart rate (bpm)	170 ± 8^‡^	161 ± 9*	148 ± 15^∗,†^
Percentage of peak heart rate (%)	91.6 ± 5.5	85.1 ± 6.1*	88.7 ± 5.4
Submaximal SBP (mm Hg)	122 ± 12	136 ± 12*	129 ± 20
Submaximal DBP (mm Hg)	58 ± 14	65 ± 11	65 ± 10

Values are significant at *P* < 0.050; *different than NW; ^†^different than OB; ^‡^different than PWS.
